# Neurotropic Tick-Borne Flavivirus in Alpine Chamois (*Rupicapra rupicapra rupicapra*), Austria, 2017, Italy, 2023

**DOI:** 10.3390/v17010122

**Published:** 2025-01-16

**Authors:** Norbert Nowotny, Maria Lucia Mandola, Isabella Monne, Zoltán Bagó, Chiara Nogarol, Alice Fusaro, Katharina Dimmel, Barbara Moroni, Lisa Guardone, Jolanta Kolodziejek, Elisa Palumbo, Gabriela Stanclova, Adi Steinrigl, Gabriele Fidler, Cristina Bertasio, Irene Bertoletti, Alessandro Bianchi, Mattia Calzolari, Paola Prati, Nadia Vicari, Angela Salomoni, Maria Francesca Priore, Federica Gobbo, Aitor Garcia-Vozmediano, Tom Loney, Ahmad Abou Tayoun, Alawi Alsheikh-Ali, Paola De Benedictis, Jeremy V. Camp, Zdenek Hubalek, Ivo Rudolf, Davide Lelli, Ana Moreno

**Affiliations:** 1 Center of Pathobiology, Department of Biological Sciences and Pathobiology, University of Veterinary Medicine Vienna, 1210 Vienna, Austria; katharina.dimmel@vetmeduni.ac.at (K.D.); jolanta.kolodziejek@chello.at (J.K.); 2 College of Medicine, Mohammed Bin Rashid University of Medicine and Health Sciences, Dubai Health, Dubai P.O. Box 505055, United Arab Emirates; tom.loney@dubaihealth.ae (T.L.); ahmad.tayoun@dubaihealth.ae (A.A.T.); alawi.alsheikhali@dubaihealth.ae (A.A.-A.); 3 Istituto Zooprofilattico Sperimentale del Piemonte, Liguria e Valle d’Aosta (IZSPLV), 10154 Torino, Italy; marialucia.mandola@izsplv.it (M.L.M.); chiara.nogarol@izsplv.it (C.N.); barbara.moroni@izsplv.it (B.M.); lisa.guardone@unipi.it (L.G.); aitor.garciavozmediano@izsplv.it (A.G.-V.); 4 Istituto Zooprofilattico Sperimentale delle Venezie (IZSVe), 35020 Legnaro, Italy; imonne@izsvenezie.it (I.M.); afusaro@izsvenezie.it (A.F.); epalumbo@izsvenezie.it (E.P.); asalomoni@izsvenezie.it (A.S.); mfpriore@izsvenezie.it (M.F.P.); fgobbo@izsvenezie.it (F.G.); pdebenedictis@izsvenezie.it (P.D.B.); 5 Institute for Veterinary Disease Control Mödling, Austrian Agency for Health and Food Safety Ltd. (AGES), 2340 Mödling, Austria; zoltan.bago@ages.at (Z.B.); gabriela.stanclova@ages.at (G.S.); adi.steinrigl@ages.at (A.S.); 6 Department of Veterinary Sciences, University of Pisa, 56124 Pisa, Italy; 7 Veterinary Practice, Reitsam, 5450 Werfen, Austria; gabi.fidler@sbg.at; 8 Istituto Zooprofilattico Sperimentale della Lombardia e dell’Emilia Romagna (IZSLER), 25124 Brescia, Italy; cristina.bertasio@izsler.it (C.B.); irene.bertoletti@izsler.it (I.B.); alessandro.bianchi@izsler.it (A.B.); mattia.calzolari@izsler.it (M.C.); paola.prati@izsler.it (P.P.); nadia.vicari@izsler.it (N.V.); davide.lelli@izsler.it (D.L.); anamaria.morenomartin@izsler.it (A.M.); 9 Center for Virology, Medical University of Vienna, 1090 Vienna, Austria; jeremy.camp@meduniwien.ac.at; 10 Institute of Vertebrate Biology, Czech Academy of Sciences, 60365 Brno, Czech Republic; zdehubalek@seznam.cz (Z.H.); rudolf@ivb.cz (I.R.)

**Keywords:** Alpine chamois, *Rupicapra rupicapra*, *Ixodes ricinus*, flavivirus, *Flaviviridae*, tick-borne encephalitis virus, louping ill virus, Spanish goat encephalitis virus, neurotropic, encephalitis

## Abstract

The European subtype of tick-borne encephalitis virus (TBEV-Eur; species *Orthoflavivirus encephalitidis*, family *Flaviviridae*) was the only tick-borne flavivirus present in central Europe known to cause neurologic disease in humans and several animal species. Here, we report a tick-borne flavivirus isolated from Alpine chamois (*Rupicapra rupicapra rupicapra*) with encephalitis and attached ticks, present over a wide area in the Alps. Cases were detected in 2017 in Salzburg, Austria, and 2023 in Lombardy and Piedmont, Italy. The virus strains exhibit 94.8–97.3% nucleotide identities to each other and are more closely related to Louping ill viruses (LIV; *Orthoflavivirus loupingi*; 90–92% identities) than to TBEV-Eur (less than 88%). The chamois-derived virus strains, tentatively termed “Alpine chamois encephalitis virus”, form a well-supported independent genetic clade with Spanish goat encephalitis virus, clearly separated from other LIV. This supports its designation as a new virus subtype with the proposed shared taxonomic name “Spanish goat and Alpine chamois encephalitis virus subtype” within the species *Orthoflavivirus loupingi*. The zoonotic potential of this newly identified virus subtype as well as its host range in other animal species including farm animals needs to be further investigated.

## 1. Introduction

The disease known as tick-borne encephalitis (TBE) was first described in 1931 by the Austrian clinician Hans Schneider in and around the city of Neunkirchen, Lower Austria [1,2]. The etiologic virus, tick-borne encephalitis virus (TBEV), was identified in 1937 during a scientific expedition to the Far East by the Russian/Soviet scientist Lev A. Zilber (reviewed in [3]). The European form of the virus was first described in 1948 independently by Gallia et al. [4] and Krejčí [5] in former Czechoslovakia (reviewed in [3,6]). Traditionally, three geographically widely distributed and partially overlapping TBEV subtypes have been recognized within the virus species *Orthoflavivirus encephalitidis*, i.e., the European subtype (TBEV-Eur), the Far Eastern subtype (TBEV-FE), and the Siberian subtype (TBEV-Sib) [7]. In addition to these main TBEV subtypes, two other subtypes, i.e., the Baikalian (TBEV-Bkl) and the Himalayan subtype (TBEV-Him), have been described recently [8,9].

The natural transmission cycle of TBEV-Eur involves small forest mammals, especially rodents and insectivores, and possibly also certain bird species, and ticks of the genus *Ixodes*, mainly *Ixodes ricinus*, as arthropod vectors [10]. Geographically, TBE is present in Europe in at least 19 countries [11] in the form of endemic foci/microfoci [12]. The number of TBE cases varies from year to year, in central Europe mainly depending on the fructification of the European beech (*Fagus sylvatica*), a major food source of rodents [13]. Due to global warming and several other drivers of TBE emergence and increased incidence in humans [14,15], the TBEV-endemic areas have expanded in recent decades, particularly to the north and west, and to higher altitudes [11,14,15,16,17,18,19]. The spread of TBEV to previously non-endemic regions has also been reported in Italy [20,21].

Beyond reservoir hosts, TBEV also infects other mammals. The infection is usually subclinical in adult ruminants such as goats, sheep, and (rarely) cows, however, they all might excrete the virus in the milk [18,22]. Besides transmission via *Ixodes* ticks, alimentary transmission of TBEV to humans has been reported through the consumption of raw milk and unpasteurized dairy products, such as cheese derived from viremic animals [18,23]. Dogs and horses are also susceptible to TBEV infection [24,25,26] but they rarely develop neurologic disease and do not apparently play a role in the disease transmission cycle [27,28,29]. Rare cases of clinical TBE were also reported from wild species of the *Caprinae* and *Capreolinae* subfamilies such as mouflon (*Ovis ammon musimon*; [30]), Alpine chamois (*Rupicapra rupicapra rupicapra*; [21]) and roe deer (*Capreolus capreolus*; [31]). Wild ungulates, such as Alpine chamois, are also investigated as possible sentinels of virus circulation as they show neutralizing antibodies against TBEV [32].

Humans are incidental and dead-end hosts and do not play a role in the maintenance of the virus in nature [16]. TBE in humans often shows a biphasic course with a viremic phase characterized by non-specific flu-like symptoms, followed by an asymptomatic period and progression to a neurologic phase characterized by meningoencephalitis, meningitis, or meningoencephalomyelitis in more than half of patients. The estimated case fatality ratio is approximately 1% [33,34]. Over the years, the application of TBE vaccines in humans has led to a significant decrease in cases in endemic areas with high vaccination rates [35,36].

Here, we report and characterize a newly identified tick-borne flavivirus subtype isolated from three independent cases of Alpine chamois (*R. r. rupicapra*) with encephalitis and attached ticks in Austria and Italy.

## 2. Materials and Methods

### 2.1. Case Histories

The case histories were documented by the hunters and veterinarians on site.

### 2.2. Postmortem and Pathohistological Investigations

The age of the Alpine chamois was estimated according to [37] on teeth eruption and by counting the number of annual incremental growth rings on the horns.

The postmortem and pathohistological investigations were carried out according to standard procedures in all cases. Of the central nervous system, sections in the area of the basal ganglia, thalamus, mesencephalon, cerebellum, pons, medulla oblongata, brain stem, and spinal cord were histologically investigated.

### 2.3. Molecular Investigations

#### 2.3.1. Case 1: Province of Salzburg, Austria, 2017

A pool of brain stem and cerebrum of the Alpine chamois designated N5/17 was homogenized with cooled Dulbecco’s Modified Eagle Medium (DMEM, Gibco, Grand Island, NY, USA) using an automated TissueLyser II (Qiagen, Redwood City, CA, USA) and 2.8 mm zirconium oxide beads (Precellys 24, Bertin Technologies, Montigny-le-Bretonneux, France), followed by centrifugation at 835× *g* for 5 min. After RNA extraction of the supernatant, using the QIAcube^®^ instrument (Qiagen) and the QIAamp Viral RNA Mini kit (Qiagen), a one-step RT-PCR assay (Qiagen) was performed using pan-flaviviral primers detecting a fragment of the NS5 gene [38]. The obtained PCR product was subjected to sequencing (Eurofins Genomics). Since the partial sequence showed the highest identity to a virus derived from a Spanish goat, the primers used by Mansfield et al. were employed in order to establish the sequence of the entire polyprotein-coding region of the chamois-derived virus [39]. Remaining sequence gaps were filled by designing oligonucleotides with the help of the Primer Designer 4 program, version 1.1 (Scientific & Educational Software, Cary, NC, USA) and applying RT-PCRs with specific primer pairs bridging the gaps based on the already obtained sequences, thus generating the complete coding sequence.

Since no ticks were attached to the chamois when shot, ticks were collected from other animals in the area in spring 2017 and typed to species. They were subjected to homogenization and nucleic acid extraction as described above. Two RT-PCR assays were applied to the tick extracts, a pan-flavivirus assay [38] and an in-house RT-PCR specific for the N5/17 sequence.

#### 2.3.2. Case 2: Region of Piedmont, Italy, 2023

Brain and lung tissues were sampled from the Alpine chamois (ID IZSPLV: 63100/2023). Seven adult ticks attached to the chamois were morphologically characterized to species level using taxonomical keys [40]. Chamois tissues and ticks were initially assessed by an RT-PCR targeting the 3′ noncoding region of the TBEV genome [41]. Briefly, samples were homogenized with 2.8 mm zirconium oxide beads (Precellys 24, Bertin Technologies, Montigny-le-Bretonneux, France) in homogenization solution (Promega Corp., Madison, WI, USA) using an automated Tissue Lyser II (Qiagen Redwood City, CA, USA), followed by centrifugation at 1000× *g* for 10 min. Nucleic acid was extracted from tick homogenates (200 μL) using The Maxwell^®^ RSC 48 automated nucleic acid purification platform and the Maxwell RSC Simply RNA Tissue kit (Promega Corp., Madison, WI, USA), following manufacturer’s instructions, while the nucleic acid from the brain and lung tissues were extracted using the All Prep DNA/RNA mini Kit (Qiagen, Valencia, CA, USA). Abundant rRNA and globin RNA were removed from 200 ng of total RNA by ligation with Ribo-Zero Plus as part of the Illumina Stranded Total RNA Prep kit (Illumina, San Diego, CA, USA). Ribodepleted samples then underwent library preparation and were sequenced on an Illumina NextSeq550 instrument using a NextSeq 500/550 Mid Output Kit v2.5 (2 × 150 bp paired-end mode) (Illumina, San Diego, CA, USA) at IZSVe. Consensus sequence of the whole genome was obtained using the CZ-ID online platform [42].

#### 2.3.3. Case 3: Region of Lombardy, Italy, 2023

Brain of the Alpine chamois [Istituto Zooprofilattico Sperimentale della Lombardia e dell’Emilia Romagna (IZSLER) id: 271964/2023] and the attached ticks were homogenized separately in PBS containing antibiotics and glycerol (10%) using an automated TissueLyser II (Qiagen, Redwood City, CA, USA) and 4.5 mm zinc plated steel beads, followed by centrifugation at 1575× *g* for 15 min. Viral nucleic acid was extracted from 200 μL supernatant using King Fisher instrument (ThermoFisher Scientific, Waltham, MA, USA) and the IndiMag Pathogen kit (Indical Bioscence, Leipzig, Germany) according to the manufacturer’s instructions. Pan-flavivirus RT-PCR [43] and TBEV RT-qPCR [41] were used as first-line diagnostic tests, considering the previous identification of TBEV in Lombardy in May 2023 in an Alpine chamois with neurological signs [21].

To obtain the whole genome sequence of the detected virus, library was prepared with the Illumina Stranded Total RNA Prep, Ligation with Ribo-Zero Plus (Illumina, San Diego, CA, USA). Sequencing was performed on Illumina Miniseq instrument using a MiniSeq Mid Output Kit (2 × 150 bp paired-end mode) (Illumina, San Diego, CA, USA). Consensus sequence of the whole genome was obtained by mapping the trimmed reads to the genome of Spanish goat encephalitis virus (SGEV; acc.no.: KP144332) using Geneious Prime 2022.2.

### 2.4. Phylogenetic Investigations on All Alpine Chamois and Tick-Derived Complete Genome Sequences

Before phylogenetic analyses, multiple alignments of a dataset of forty-four 10,245 nt long complete polyprotein coding nucleotide sequences of TBEV, louping ill virus (LIV), and LIV-like viruses available in GenBank were performed using the DNASTAR navigator (version 13.3.0.57) with the ClustalW algorithm. A maximum likelihood phylogenetic tree based on whole genome sequences was constructed using IQtree (version 2.6) [44] and Model Finder software (R2023b) to determine the best model. A reduced dataset of twelve sequences including the Austrian and Italian chamois-derived viruses, the SGEV, and the TBEV and LIV reference strains was used to investigate the genetic relationships throughout the complete genome by generating similarity diagrams of these genomes using SSE v1.4 with a sliding window of 800 and a step size of 10 nt.

### 2.5. Further Molecular Studies

The same dataset of sequences was also used for molecular analysis of the polyprotein to evaluate the amino acid mutations detected in the sequences investigated in this study in comparison to TBEV strains, LIV, and LIV-like sequences. In addition, structure information of the E protein was defined based on an ESPript algorithm (http://espript.ibcp.fr (accessed on 15 April 2024)) [45] and the E protein structure of LIV (PDB acc.no. 6J5C).

### 2.6. Virus Isolation

#### 2.6.1. Case 1

A 0.02 mL amount of clarified chamois brain homogenate was inoculated intracerebrally (i.c.) in each of 11 suckling ICR mice. The brains of those suckling mice that succumbed were homogenized and inoculated i.c. into four 23-day-old ICR mice (0.02 mL), and also subcutaneously (s.c.) into four 23-day-old ICR mice (0.2 mL each). Thereafter, a virus neutralization test was performed with the isolate on Vero E6 cells using a mouse antiserum against TBEV reference strain Hypr.

#### 2.6.2. Case 2

A 0.5 mL amount of clarified chamois brain homogenate was inoculated on confluent monolayers of Vero-E6 and BHK-21 cells (ATCC^®^ cat.n. CRL 1586 and CCL-10, respectively), incubated at 37 °C with 5% CO_2_ and checked daily for 7 days for the presence of cytopathic effects (CPE). After three blind passages, virus isolation was assessed through immunofluorescence staining. Briefly, cells were fixed with 4% paraformaldehyde for 15 min at room temperature, washed three times with PBS, and permeabilized with 0.1% Triton X-100 in PBS for 5 min. Cells were then incubated with the primary anti-dsRNA antibody (SCICONS cat.n. 10010200) overnight at 4 °C and then with an anti-mouse IgG (H + L) Cross-Adsorbed Secondary Antibody, Alexa Fluor™ 568 (Thermo Fisher Scientific, cat.n. A-11004) for 2 h at room temperature. Cells were then observed using a fluorescent microscope (Leica DMi8). After three blind passages, both inoculated cell lines were also evaluated for the presence of viral RNA by RT-qPCR: Total RNA was isolated from cell culture supernatant by using the All Prep DNA/RNA mini Kit (Qiagen, Valencia, CA, USA), as per the manufacturer’s protocol, followed by RT-qPCR targeting the 3′ noncoding region of the TBEV genome [41].

#### 2.6.3. Case 3

Thirteen 1–2 days old suckling mice were i.c. inoculated with 0.01 mL of clarified chamois brain homogenate. The brains of those suckling mice that succumbed to infection were homogenized and inoculated into 24-well plates in confluent monolayer of Vero-E6 cells, incubated at 37 °C with 5% CO_2_, and checked daily for 7 days for the presence of cytopathic effects (CPE). A minimum of three blind passages were performed. The successful viral isolation was assessed by testing the culture with immunoperoxidase staining (IPMA) using anti-TBEV monoclonal antibodies (mAbs) produced at IZSLER and by RT-qPCR.

## 3. Results

### 3.1. Case Histories

#### 3.1.1. Case 1: Province of Salzburg, Austria, 2017

**Figure 1:**
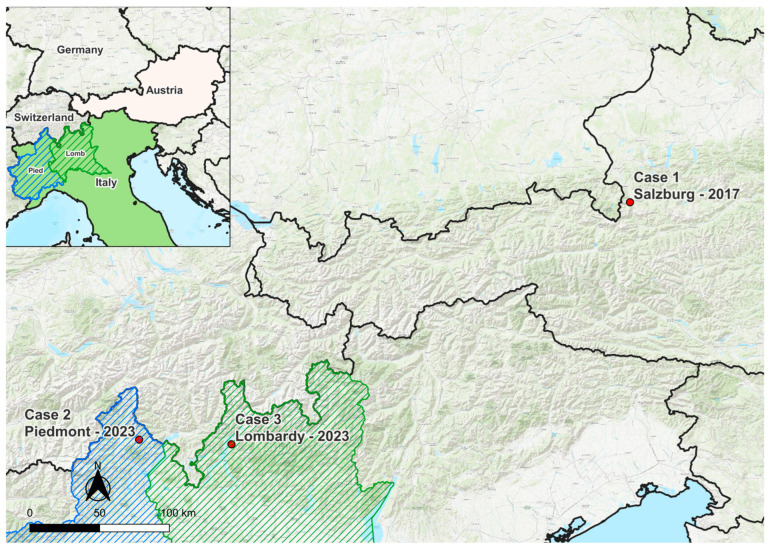
Location of the three cases of Alpine chamois encephalitis virus infections in Austria and northern Italy, indicated by red dots. The straight line distances between the cases were 390 km [case 1 (Salzburg) and 2 (Piedmont)], 334 km [case 1 (Salzburg) and 3 (Lombardy)], and 66 km [case 2 (Piedmont) and 3 (Lombardy)], respectively.

On 11 February 2017, a 7-year-old male Alpine chamois (*R. r. rupicapra*) was shot by a hunter in the Bluntau Valley (47.584° N 13.150° E) near the village of Golling an der Salzach in the Austrian federal state of Salzburg at 1700 m above sea level (a.s.l.) (Figure 1). The chamois showed unusual behavior consistent with neurologic signs (unsteady gait, torticollis). No ticks were attached to the chamois.

#### 3.1.2. Case 2: Region of Piedmont, Italy, 2023

A 5-year-old male Alpine chamois in poor body condition was found dead on 18 May 2023 in the Verbania municipality, about 1 km west of Malesco (Piedmont, Italy, 46.126° N 8.486° E) within the Val Grande National Park at 761 m a.s.l. (Figure 1). The carcass was transported to the Istituto Zooprofilattico Sperimentale Piemonte, Liguria and Valle d’Aosta in Turin (“IZSPLV”, Italy) to determine the cause of death within the framework of the regional passive surveillance plan for wildlife. The seven adult ticks attached to the chamois were identified as *I. ricinus* (4 engorged females and 2 males) and *Haemaphysalis sulcata* (1 female).

#### 3.1.3. Case 3: Region of Lombardy, Italy, 2023

On 4 September 2023, a 5-year-old male Alpine chamois was found dying with neurologic signs of ataxia, incoordination, and muscle tremors, at the Artesso lake, Sueglio municipality, Lombardy, Italy (46.097° N 9.343° E) at 1200 m a.s.l. (Figure 1). The chamois had been spotted in the area with abnormal escape behavior and were euthanized and conferred to IZSLER for necropsy as part of the regional surveillance plan of wild fauna in Lombardy. The chamois had a high tick burden. Specifically, 23 ticks were collected from the head, all identified as *I. ricinus* (10 adult females, 5 adult males, 6 nymphs, and two larvae).

### 3.2. Postmortem and Pathohistological Results

#### 3.2.1. Case 1

**Figure 2:**
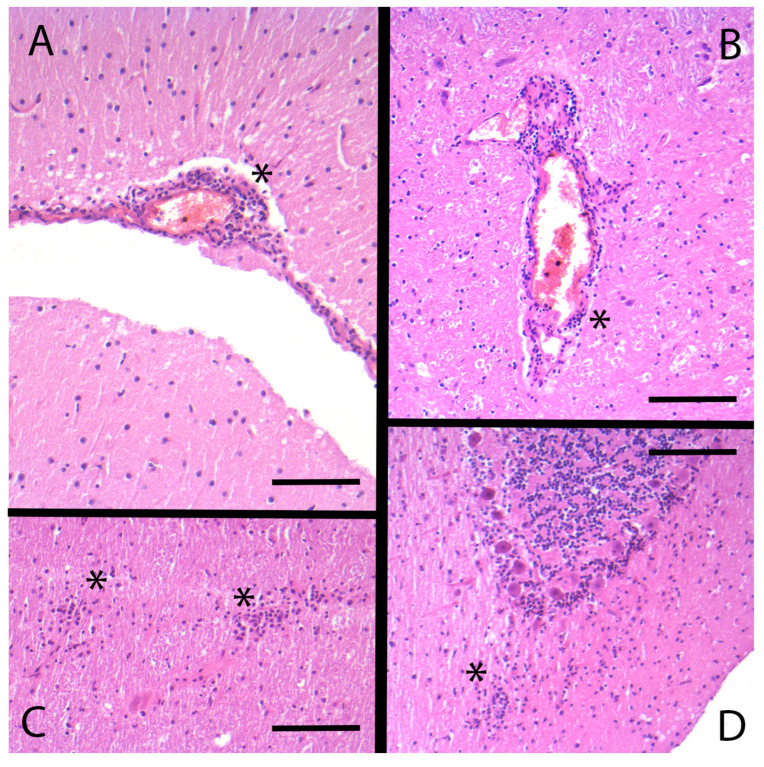
Histological sections from the brain of case 1. (**A**) Nonpurulent leptomeningitis: Slight lymphoplasmohistiocytic infiltrate (*); (**B**–**D**) Nonpurulent encephalitis; (**B**): Slight lymphoplasmohistiocytic perivascular infiltrate (*); (**C**) Two glial nodules in brain stem (*); (**D**) Glial shrubbery in the cerebellum (*). Microphoto, H&E, bar (**A**) = 100 µm, bars (**B**–**D**) = 180 µm.

Gross pathology revealed no remarkable findings aside from intestinal and pulmonary parasitic infestation. The chamois showed a slight non-suppurative leptomeningoencephalitis with perivascular infiltrates consisting of lymphocytes, plasma cells and histiocytes, and multiple glial nodes as well as slight acute perivascular hemorrhages (Figure 2), the latter most likely was a result of the shot.

#### 3.2.2. Case 2

At postmortem examination, the chamois was found to be severely cachectic with mild bilateral conjunctivitis and showed incomplete molt. Chewing lice (mallophaga) and ticks were present. Verminous pneumonia, splenomegaly, and generalized lymphadenopathy were also present. Due to the advanced decomposition status, it was not possible to perform a histopathological examination of the brain and other organs.

#### 3.2.3. Case 3

Postmortem examination revealed poor general condition with massive tick infestation. Histological examination of the encephalon (sections of the cerebral cortex and cerebellar cortex) revealed minimal information with mild, multifocal marginalization of leukocytes in the vessels. Chronic, severe, and diffuse lympho-histiocytic meningoencephalitis with perivascular cuffs and glial nodules in the pons section were detected.

### 3.3. Molecular Diagnosis

#### 3.3.1. Case 1

The complete coding sequence (10,766 nt) of the virus labeled N5-17/chamois/Austria/2017 was determined (GenBank Accession MG243699). Fifty-seven ticks (43 female and 14 male *I. ricinus* individuals) were collected in spring 2017 from other animals in the region. No specific nucleic acid was detected in them, neither by pan-flavivirus RT-PCR nor by N5/17-specific RT-PCR.

#### 3.3.2. Case 2

Almost complete viral genome sequences (10,432 nt) were obtained from the brain tissue of the chamois as well as from a female *I. ricinus* tick (GenBank Accession PP795456 and PP795457).

#### 3.3.3. Case 3

Pan-flavivirus RT-PCR and TBEV RT-qPCR showed positivity in the brain of the chamois and in 10 of 23 attached ticks (seven adult females, two males, and one larva). Almost complete flavivirus sequences were obtained from the brain sample of the chamois and from a pool of the attached ticks. The chamois- and tick-derived sequences were identical to each other (GenBank Accession PP782055, PP782054 and PP782056).

### 3.4. Molecular Phylogeny on Alpine Chamois and Tick-Derived Complete Genome Sequences

**Figure 3:**
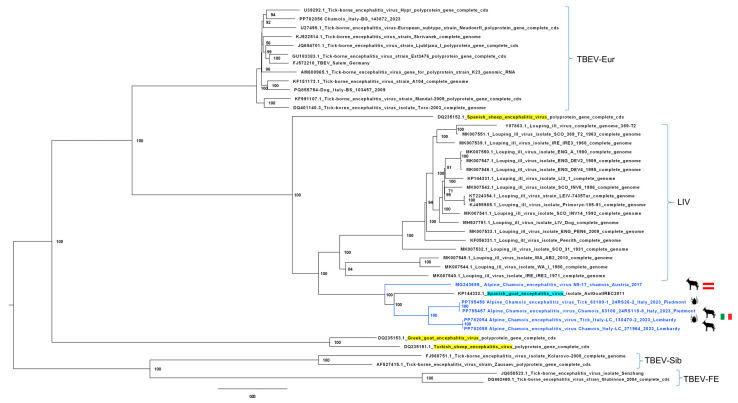
Maximum likelihood phylogenetic tree of the complete genomes of Alpine chamois encephalitis viruses and other TBEV, LIV, and LIV-like sequences constructed using the IQtree software and the GTR + F+I + G4 model according to BIC and a bootstrap of 1000 replicates. Sequences investigated in this study are shown in blue. The sequences of Spanish sheep encephalitis virus, Greek goat encephalitis virus, and Turkish sheep encephalitis virus are highlighted in yellow, and the sequence of Spanish goat encephalitis virus is highlighted in turquoise.

An ML phylogenetic tree of the complete genome sequences placed the five chamois-derived flavivirus sequences into a single monophyletic group (similarity ranging from 94.75% to 97.31%) (Figure 3). This group, which includes SGEV, identified in a goat in Spain in 2011 [34], shares a common ancestor with some members of the clade of the *Orthoflavivirus loupingi* species (namely Louping ill virus (LIV), British subtype (LIV-Brit), and Irish subtype (LIV-Ir)), but clusters separately from the Spanish subtype (LIV-Spain = Spanish sheep encephalitis virus, SSEV), Turkish sheep encephalitis virus subtype (TSEV), and Greek goat encephalitis virus subtype (GGEV) (https://ictv.global/report/chapter/flaviviridae/flaviviridae/orthoflavivirus (accessed on 15 March 2024); Figure 3).

**Figure 4:**
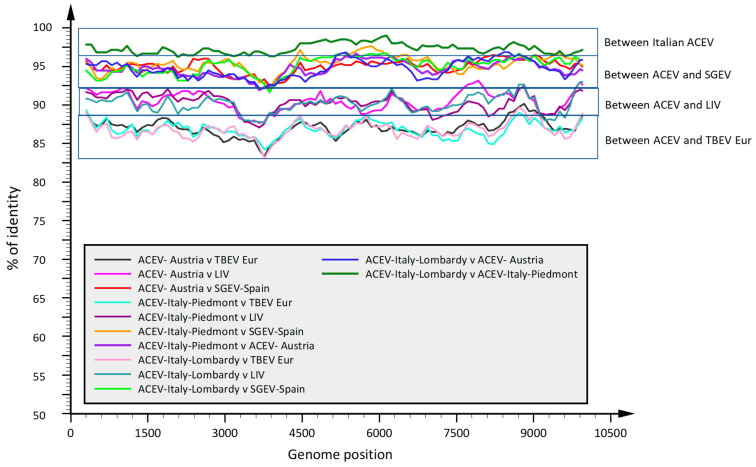
SSE similarity plots over the whole genome sequences between the Alpine chamois encephalitis viruses (ACEV) from Austria and Italy, the Spanish goat encephalitis virus (SGEV), and the reference strains of tick-borne encephalitis virus European subtype (TBEV-Eur) and Louping ill virus (LIV). Tick and chamois virus sequences for Piedmont and Lombardy are tagged as a group. Similarity plots were generated using SSE version 1.2 using a sliding window of 600 and a step size of 100 nucleotides. The percentage of identities (*y*-axis) between 50 and 100% is shown over the whole genome alignment (*x*-axis).

The newly characterized virus subtype, provisionally named “Alpine chamois encephalitis virus; ACEV” show a similarity ranging from 94.77 to 97.32% compared to SGEV, as well as from 90.31 to 90.62% and from 86.73 to 86.97% compared to LIV (accession number Y07863) and TBEV-Eur (accession number U27495) reference strains, respectively. The whole genome identity of ACEV in this study was compared with SGEV and the reference strains TBEV-Eur and LIV, and sequence identity calculation was made using DNASTAR software using the default parameters. The SSE graph shows a greater identity between the two Italian ACEVs compared to the Austrian strain, and a greater identity of the Italian and Austrian ACEVs with SGEV with respect to the LIV and TBEV-Eur reference strains (Figure 4).

### 3.5. Results of Further Molecular Studies

**Figure 5:**
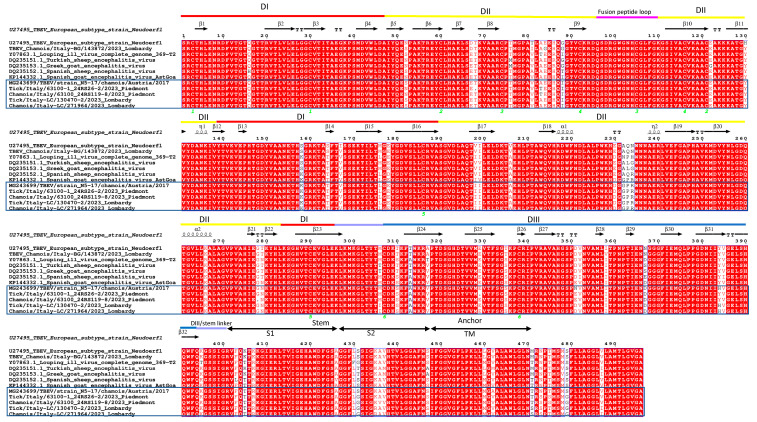
Structural alignment of the E protein. The secondary structure elements are defined based on an ESPript algorithm and are labeled as in a previous report on the LIV structure (PDB no. 6J5C) and are presented on top (Spiral lines indicate helices, arrows represent β strands and TT letters turns). The Arabic numerals 1–6 indicate cysteine residues that pair to form disulfide bonds. Lines above the sequences indicate domains I (red), II (yellow), and III (blue), as well as the fusion peptide loop (pink) and the dI/dIII and dIII/stem linkers (purple). Austrian and Italian ACEV strains are shown in a box.

The flavivirus envelope protein is located on the virus surface and is involved in receptor recognition for virus entry and subsequent virus-host membrane fusion. The ectodomain of protein E (residues 1 to 401) is divided into three domains (DI, DII, and DIII) arranged linearly. DII includes a fusion peptide within a loop between two beta strands that is well-conserved in all flaviviruses. DIII is an Ig-C-like domain and includes two linker regions, which are also well-conserved in all sequences. Few amino acid differences are detected when we compare the E protein sequences of the three chamois-derived viruses with TBEV, LIV, and the more related SGEV [39]. The aa mutations are found across the whole protein, some common to all three viruses but others characteristic of one or two of them (Figure 5). Marin et al. [46] identified unique tri-peptide motifs (aa position 232 to 234 of E protein) for a number of different tick-borne flaviviruses, including LIV and LIV-Spain, which were asparagine–proline–histidine (NPH) and alanine–glutamine–arginine (AQR), respectively. In this study, the unique motif of glycine–proline–arginine (GPR) was confirmed by the five ACEV and SGEV [35]. Also, the hexa- and penta-peptide genetic marker EHLPTA (aa position 207 to 212) and DSGHD (aa position 320 to 324) of the E protein, which defines the TBE group flaviviruses were conserved in all representative amino acid sequences (Figure 5).

### 3.6. Virus Isolation

#### 3.6.1. Case 1

Three of 11 i.c.-inoculated suckling ICR mice died 8 days post-infection (d.p.i.). In the following passage, their brain homogenates killed four of four 23-day-old ICR mice inoculated i.c. 4–5 d.p.i. and also four of four 23-day-old ICR mice inoculated s.c. 8–10 d.p.i. Virus neutralization of the isolate in Vero E6 cells against mouse Hypr antiserum revealed a log neutralization index of 3.5 which identified this agent as TBE group virus. RT-PCR amplification products and sequencing results confirmed that the inoculated isolate N5/17 was detectable in the brain samples of three-week-old laboratory mice.

#### 3.6.2. Case 2

ACEV was successfully isolated at the third blind passage in both BHK-21 and VERO E6 cells. While the virus produced a clear CPE in BHK-21 cells, VERO E6 cells did not show CPE, but viral isolation was confirmed through specific immunofluorescence staining, showing the presence of dsRNA derived from viral infection. In both cell lines, the presence of viral RNA was also confirmed by RT-qPCR. The virus isolated from BHK-21 was submitted to the European Virus Archive platform as ACEV (Alpine Chamois Encephalitis Virus) Ref-SKU: 025V-05970.

#### 3.6.3. Case 3

Six out of thirteen i.c.-inoculated suckling mice died 5–6 d.p.i. Brain homogenate inoculated onto confluent monolayers of Vero E6 cells caused mild CPE starting from the second passage at 5 days post-inoculation. Virus isolation was confirmed by IPMA using anti-TBEV mAbs and comparing and verifying the increase in cycle threshold values obtained by real-time RT-PCR in the brain homogenate and different samples of infected cell passages.

## 4. Discussion

In this paper, we describe a newly identified neurotropic tick-borne flavivirus subtype isolated from Alpine chamois (*R. r. rupicapra*), tentatively designated ACEV, which was detected in three separate provinces, one in Austria and two in Northern Italy, up to 390 km (Figure 1) and more than 6 years apart. Interestingly, also the time of the year when the infected diseased/dead chamois were found showed a wide range. i.e., February (case 1), May (case 2) and September (case 3), as well as the altitude they were found, i.e., 761 m a.s.l. (case 2), 1200 m (case 3) and 1700 m (case 1), respectively. The cases differed also in their tick infestation: Case 1 was shot on 11 February at 1700 m a.s.l, i.e., at an altitude in which there is constant frost in winter. As expected at that time of the year, no ticks were attached to the chamois, and the infection must have occurred several weeks or even months earlier. This possibly suggests a different pathogenesis of the disease, which needs to be further investigated. In cases 2 and 3, ticks were attached to the chamois, and in both cases, *I. ricinus* ticks proved to be ACEV-nucleic acid positive, suggesting that ACEV is a tick-borne pathogen. *I. ricinus* was shown to occur in altitudes of more than 1800 m [47], while *H. sulcata* was so far only reported from the Maritime Alps, influenced by maritime climate, and not in an Alpine environment reported here [47,48].

Until now, TBEV-Eur was the only tick-borne encephalitis-associated flavivirus present in central Europe and Italy. The ACEV isolates from the different areas share nucleotide identities of (only) 94.75–97.31%, which suggests divergence over a relatively long evolutionary time, considering the rate of evolution of other zoonotic flaviviruses. Kutschera and Wolfinger, who included the Austrian chamois-derived sequence in their comparative molecular analyses of more than 220 TBEV strains, noted that this strain appears ancestral to all other European TBEV clades [49].

While the newly identified viruses reported here are related to LIV and distantly related to TBEV-Eur strains, they comprise a monophyletic clade with SGEV and a sister group to LIV (Figure 3). SGEV was identified in September 2011 in a herd of 70 Bermeya goats, an endangered breed of Asturian goats, in Northern Asturias, Northwestern Spain. During that outbreak, 18 goats showed neurological signs and died over a 4-month period [50,51]. Over three years, the herd mortality rate reached 100% [50]. The brain lesions resembled those of louping ill, a disease of sheep and other animal species known since the eighteenth century that is widely distributed on the British Isles and closely associated with the presence of its arthropod vector, the hard tick *I. ricinus* [52]. Subsequently, whole genome sequencing and phylogenetic analysis showed that SGEV is significantly different [39] from LIV and LIV-Spain, another tick-borne virus detected in 1987 in sheep in the Basque region of northern Spain [46,53]. Nevertheless, experimental infection of sheep with SGEV revealed that they are equally susceptible to this virus, leading to encephalitis and death, with clinical presentation, pathogenesis, and lesion morphology very similar to those of louping ill [54]. Of note, the vaccination of goats with a LIV vaccine conferred effective protection against SGEV, providing crucial information on possible control strategies in animals [55]. The 2011–2012 outbreak of SGEV in Northwestern Spain remains the only published SGEV outbreak.

**Figure 6:**
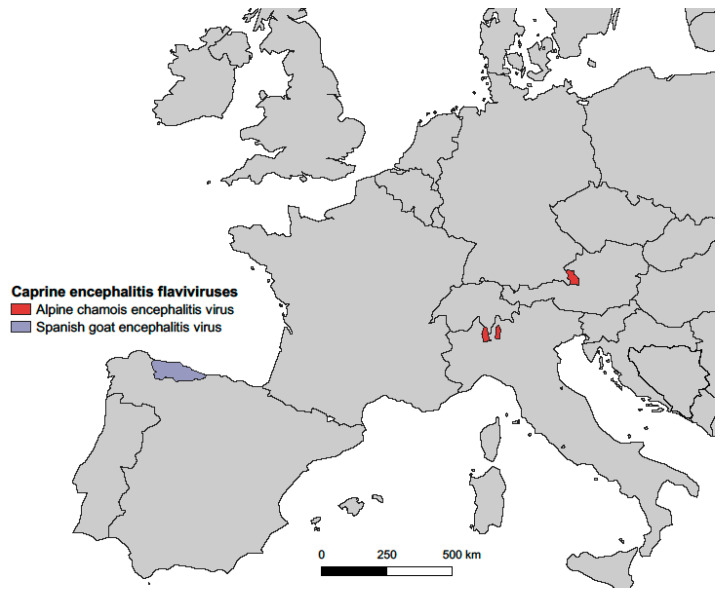
Map of Europe. Location of the three cases of Alpine chamois encephalitis virus infections in Austria and Northern Italy and approximate location of the cases of Spanish goat encephalitis virus infections in the Principality of Asturias (capital: Oviedo) in Northwestern Spain are displayed. The approximate straight line distance between cases of Alpine chamois encephalitis virus and Spanish goat encephalitis virus infections (for the latter, the exact location was not published) is between 1170 km (Piedmont case, Oviedo) and 1550 km (Salzburg case, Oviedo). The base map shapefile was obtained from the Geographic Information System of the EU Commission via the Eurostat webpage—the 2021 version at 20 M resolution showing NUTS level 3 regions—and annotated in QGIS v.3.4.15.

Molecular analysis showed a high conservation of the E protein with only 10 amino acid changes among ACEVs and a high similarity with SGEV, with which it shares the distinctive 3 amino acid GPR motif in the E protein. The similarities between ACEV and SGEV are remarkable given that they occurred more than 1100 km and several years apart (Figure 6) in different ecological environments and affected different mammal species, though both are members of the *Caprinae* subfamily. Due to the close genetic relationship of ACEVs with SGEV the shared taxonomic name “Spanish goat and Alpine chamois encephalitis virus (SGACEV) subtype” is proposed. The paper will be submitted to the *Flaviviridae* study group of ICTV for final taxonomic classification and designation.

Finally, the zoonotic potential of this virus is not clear yet and has to be investigated. TBE in humans is usually diagnosed serologically. Commonly, TBEV IgM and IgG antibodies are evaluated in serum or cerebrospinal fluid simultaneously. Alternatively, a ≥4-fold increase in TBEV-specific IgG antibodies within 2–4 weeks establishes the diagnosis. However, serologic cross-reactions between flaviviruses may hamper the diagnosis [24] as well as previous vaccinations against TBEV. Molecular detection of TBEV nucleic acid is rarely successful in routine diagnosis of human TBE due to delayed symptom onset relative to the short viremic phase. The Center of Virology of the Medical University of Vienna is the Austrian reference laboratory for flavivirus infections, and despite a high number of serologically diagnosed TBE cases in Austria [56], fewer than 12 cases were PCR-positive in blood or cerebrospinal fluid samples in the last 24 years (although not all confirmed cases were tested by PCR). All PCR-positive cases were confirmed to be the TBEV-Eur subtype by sequencing. However, we noted that our RT-qPCR [37] would likely detect ACEV as well. More importantly, as we demonstrated herein, TBEV-specific serological tests also cross-react with ACEV. It is, therefore, unknowable if any human cases of TBE in the regions described here were due to infection with ACEV. Noteworthily, in most of the European mountain ranges, Alpine chamois may share alpine and sub-alpine meadows with livestock in late spring and summer, highlighting possible transmission risks of ACEV to sheep and goats and to humans through unpasteurized dairy products. Also, ACEV killed not only i.c. inoculated suckling mice but also s.c. inoculated 23-day-old mice, possibly indicating a higher pathogenicity, which, besides several other aspects of this newly identified virus subtype, needs to be further investigated.

## 5. Future Research Directions

In this paper, we described, characterized, and isolated three viruses of a newly identified neurotropic tick-borne flavivirus subtype in chamois in three different regions of the Alps. However, several public and animal health as well as research questions still need to be addressed. This could be achieved by pan-central European efforts involving researchers from all countries with Alpine zones. Increased clinical, virological, and serological surveillance of chamois and other wild and captive ruminants, such as goats and sheep in the Alps, should be performed, as well as collection and testing of ticks in this (large) area. Since from all three reported cases, ACEV was successfully isolated, cross-neutralization tests can be carried out with the other *Orthoflavivirus loupingi* and *Orthoflavivirus encephalitidis* subtypes in order to obtain a better understanding of the serologic relationships between them. To enable this also for other researchers, we deposited the cell-culture isolate of ACEV in the European Virus Archive platform.

## Data Availability

All sequences determined during this study are available in the GenBank database under the accession numbers provided at Section 3.3. Also, the cell-culture isolate of ACEV is available from the European Virus Archive platform.
